# Supplementary material for: Causal Relationship between Aging and Anorexia Nervosa: A White-Matter-Microstructure-Mediated Mendelian Randomization Analysis
Source: Biomedicines. 2024 Aug 16;12(8):1874. doi: 10.3390/biomedicines12081874 (PMC11351342; doi:10.3390/biomedicines12081874)
Supplement: Supplementary file 1 [file biomedicines-12-01874-s001.zip › Supplementary Figure/Figure S9/Leaveoneout plot of MR analysis for ubm-b-1956 to AN.pdf]

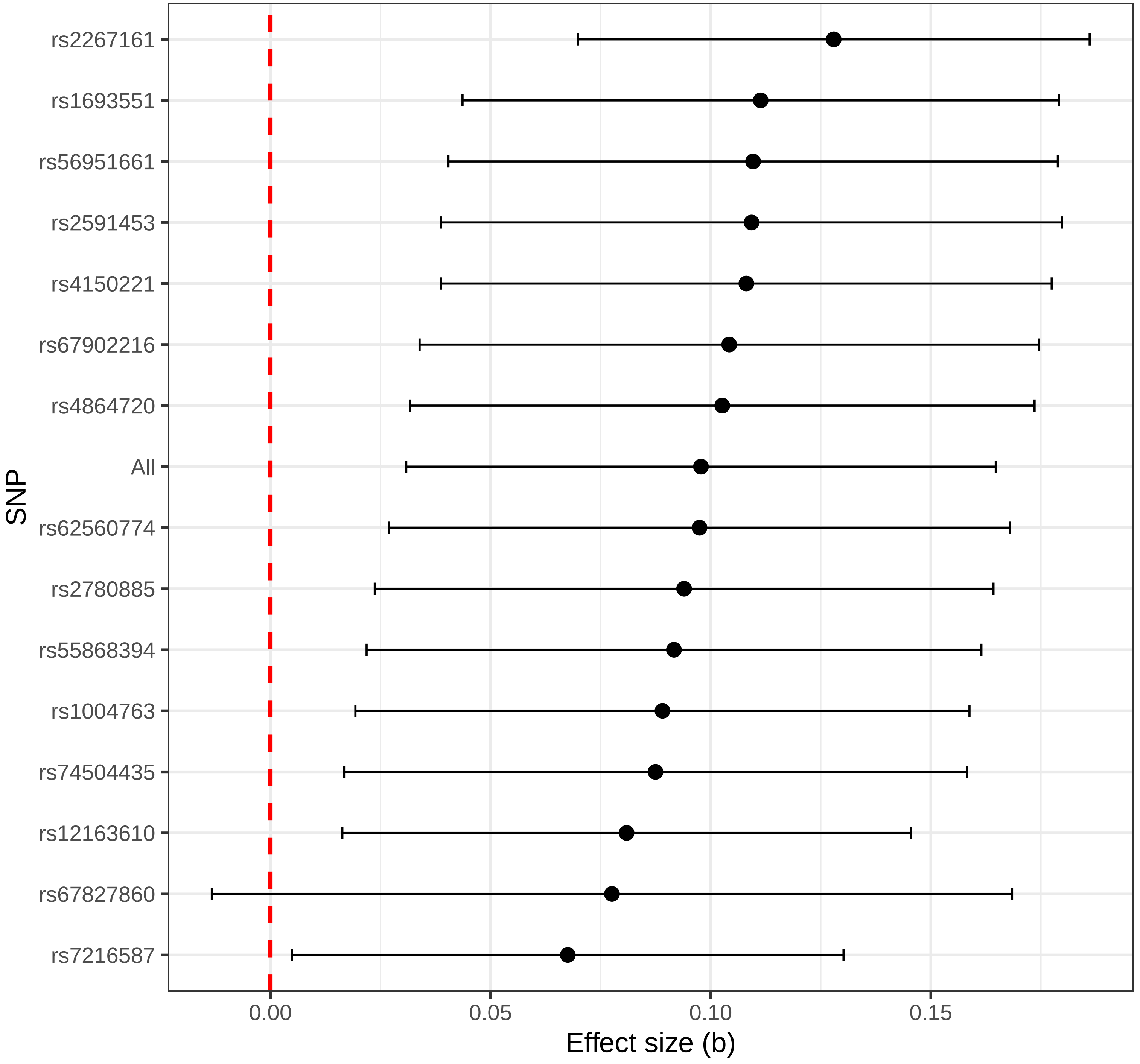

rs2267161
rs1693551
rs56951661
rs2591453
rs4150221
rs67902216
rs4864720
All
rs62560774
rs2780885
rs55868394
rs1004763
rs74504435
rs12163610
rs67827860
rs7216587
SNP
0.00
0.05
0.10
0.15
Effect size (b)
